# Preventing smoking during pregnancy: the importance of maternal knowledge of the health hazards and of the treatment options available*

**DOI:** 10.1590/S1806-37132015000004482

**Published:** 2015

**Authors:** André Luís Bertani, Thais Garcia, Suzana Erico Tanni, Irma Godoy

**Affiliations:** São Paulo State University, Botucatu School of Medicine, Department of Clinical Medicine, Botucatu, Brazil. Pulmonology Division, Department of Clinical Medicine, Botucatu School of Medicine, São Paulo State University, Botucatu, Brazil; São Paulo State University, Botucatu School of Medicine, Botucatu Hospital das Clínicas, Botucatu, Brazil. Botucatu Hospital das Clínicas, Botucatu School of Medicine, São Paulo State University, Botucatu, Brazil; São Paulo State University, Botucatu School of Medicine, Department of Internal Medicine, Botucatu, Brazil. Pulmonology Division, Department of Internal Medicine, Botucatu School of Medicine, São Paulo State University, Botucatu, Brazil; São Paulo State University, Botucatu School of Medicine, Department of Internal Medicine, Botucatu, Brazil. Pulmonology Division, Department of Internal Medicine, Botucatu School of Medicine, São Paulo State University, Botucatu, Brazil

**Keywords:** Pregnancy, Health knowledge, attitudes, practice, Smoking, Mass media, Smoking cessation, Primary prevention

## Abstract

**OBJECTIVE:**

To examine the pattern of tobacco use and knowledge about tobacco-related diseases, as well as to identify popular types of electronic media, in pregnant women, in order to improve strategies for the prevention or cessation of smoking among such women.

**METHODS:**

A cross-sectional study involving 61 pregnant women, seen at primary care clinics and at a university hospital, in the city of Botucatu, Brazil. For all subjects, we applied the Hospital Anxiety and Depression Scale. For subjects with a history of smoking, we also applied the Fagerström Test for Nicotine Dependence, and we evaluated the level of motivation to quit smoking among the current smokers.

**RESULTS:**

Of the 61 pregnant women evaluated, 25 (40.9%) were smokers (mean age, 26.4 ± 7.4 years), 24 (39.3%) were former smokers (26.4 ± 8.3 years), and 12 (19.8%) were never-smokers (25.1 ± 7.2 years). Thirty-nine women (63.9%) reported exposure to passive smoking. Of the 49 smokers/former smokers, 13 (26.5%) were aware of the pulmonary consequences of smoking; only 2 (4.1%) were aware of the cardiovascular risks; 23 (46.9%) believed that smoking does not harm the fetus or newborn infant; 21 (42.9%) drank alcohol during pregnancy; 18 (36.7%) reported increased cigarette consumption when drinking; 25 (51.0%) had smoked flavored cigarettes; and 12 (24.5%) had smoked a narghile. Among the 61 pregnant women evaluated, television was the most widely available and favorite form of electronic media (in 85.2%), as well as being the form most preferred (by 49.2%).

**CONCLUSIONS:**

Among pregnant women, active smoking, passive smoking, and alternative forms of tobacco consumption appear to be highly prevalent, and such women seem to possess little knowledge about the consequences of tobacco use. Educational programs that include information about the consequences of all forms of tobacco use, employing new and effective formats tailored to this particular population, should be developed, in order to promote smoking prevention and cessation among pregnant women. Further samples to explore regional and cultural adaptations should be evaluated.

## Introduction

Although the prevalence of smoking in the general population is well known, few studies have addressed smoking in pregnant women. In the United States, the prevalence of maternal smoking during pregnancy is estimated to be 25%.^(^ 1 ^)^ A study evaluating pregnant women seen at primary care units in the state of São Paulo, Brazil, showed that 19.2% actively smoked (during pregnancy and breastfeeding); 28.2% were active and passive smokers; and 16.8% were exposed to passive smoking only.^(^ 2 ^)^ In 2011, another study, conducted in the Brazilian state of Rio Grande do Sul, evaluated anthropometric measurements of neonates born to 2,484 women and showed that, during pregnancy, 23.3% of those women had smoked and 28.9% had been continuously exposed to secondhand smoke.^(^ 3 ^)^

Anti-smoking policies in Brazil are quite advanced. The tobacco industry is required to label packages with warnings about the health consequences of smoking, and there are laws prohibiting smoking in collective and indoor environments. Direct and indirect merchandising and sponsorship by the tobacco industry are also prohibited.^(^ 4 ^-^ 6 ^)^ However, the industry continues to employ strategies such as adding flavors to conventional cigarettes to change the taste and smell, in order to recruit new smokers from among women and adolescents. It should be borne in mind that flavored cigarettes and alternative forms of tobacco deliver all of the chemicals found in conventional cigarettes,^(^ 7 ^)^ and the prevalence of the use of such products remains unknown.

The study and analysis of pregnant women and smoking, as well as of the characteristics of nicotine addiction and its adverse health consequences, are fundamental to developing tools for the prevention and treatment of smoking in this population. There have been few studies evaluating knowledge of the hazards of smoking among pregnant women in Brazil,^(^ 2 ^,^ 3 ^,^ 8 ^)^ and none have evaluated technology preferences with the objective of designing interventions targeting such women. Therefore, the main objective of the present study was to examine the pattern of tobacco use and knowledge about tobacco-related diseases, as well as to identify the preferred forms of electronic media, in pregnant women, in order to improve strategies for promoting smoking prevention and cessation among such women.

## Methods

### Subjects

Between January and July of 2012, we recruited 61 pregnant women from among those attending routine appointments at the public prenatal care clinic of a university hospital and at primary care clinics in the city of Botucatu, Brazil. The study was approved by the Research Ethics Committee of the São Paulo State University Botucatu School of Medicine, also in the city of Botucatu. All participants (or their parents or legal guardians) gave written informed consent. 

### Study design and procedures

This was a descriptive cross-sectional study. The only exclusion criterion was refusal to participate. An investigator approached each pregnant woman while she was waiting to be seen and explained the aims of the study. The investigator was not a member of the health care team responsible for the treatment of the women recruited and visited the site only to conduct the interviews. All of the women invited agreed to participate and were directed to a room to be interviewed, in order to complete the study questionnaires. The interviews were conducted face-to-face, the investigator using a questionnaire developed specifically for the present study. The pregnant women freely answered questions about active and passive smoking, smoking habits during social activities, consumption of alcohol, use of alternative forms of smoking, and knowledge about the health hazards of smoking in general, as well as about the adverse health consequences of smoking during pregnancy, not only for the mother but also for the fetus and the newborn infant. In addition, all of the women completed the Hospital Anxiety and Depression Scale (HADS)^(^ 9 ^)^; those who were smokers or former smokers took the Fagerström Test for Nicotine Dependence^(^ 10 ^)^; and we assessed the motivational stage of change (level of readiness to quit smoking) using the model devised by DiClemente & Prochaska.^(^ 11 ^)^ All personal information was kept confidential.

### Statistical analyses

We used the chi-square test to compare the proportions and ANOVA with Tukey's test to compare means. Both evaluations were performed with a power of 80% and a level of significance of 5%.

## Results

Of the 61 pregnant women evaluated, 25 (40.9%) were smokers (mean age, 26.4 ± 7.4 years), 24 (39.3%) were former smokers (26.4 ± 8.3 years), and 12 (19.8%) were never-smokers (25.1 ± 7.2 years). The main characteristics of those three groups are presented in Table 1. Most of the women were married and were smokers or former smokers. Only 7 women (11.5%) were over 36 years of age. The predominant level of education was ≤ 9 years of schooling. Most of the women interviewed reported having been exposed to passive smoking, the prevalence of such exposure being highest among the smokers and never-smokers. Of the 25 smokers, 12 (49.2%) reported that their husband smoked, and 4 (14.7%) reported that at least one member of the household smoked. Abortion due to fetal malformation was reported only by women who were smokers or former smokers. Of the 18 women (29.7%) who had husbands who did not smoke, 10 (54.5%) were former smokers.

**Table 1 -:** Characteristics of the 61 pregnant women evaluated.

Variable	Smokers	Former smokers	Never-smokers
n (% of the sample as a whole)	25 (40.9)	24 (39.3)	12 (19.8)
Mean age (years), mean ± SD	26.4 ± 7.4	26.4 ± 8.3	25.1 ± 7.2
Married, n (%)	16 (64.0)	13 (54.2)	7 (58.3)
Level of education (years of schooling), n (%)			
≤ 9	14 (56.0)	10 (41.7)	4 (33.3)
10-12	8 (32.0)	9 (37.5)	7 (58.3)
> 12	3 (12.0)	5 (20.8)	1 (8.4)
Passive smoking, n (%)	18 (72.0)	11 (45.8)*	10 (83.3)
History of abortion, n (%)	5 (20.0)	7 (29.2)	4 (33.3)
Abortion due to fetal malformation, n (%)	3 (12.0)	2 (8.3)	0 (0.0)

* p = 0.049 vs. smokers and never-smokers.

Among the 49 pregnant women who were smokers or former smokers, the age at smoking initiation ranged from 9 years to 25 years. Of the 8 women (16.3%) who had started smoking by 12 years of age, 7 (87.5%) were current smokers (p = 0.023). In contrast, we found no association between the age at smoking initiation and current smoking among the women who had started smoking after 12 years of age. The proportional distribution of the use of alternative forms of tobacco consumption-flavored cigarettes, clove cigarettes, water pipe (narghile), and electronic cigarettes-by the smokers and former smokers is presented in Table 2.

**Table 2 -:** Alternative forms of tobacco consumption during pregnancy, by smoking status.

Variable	Smokers	Former smokers
	(n = 25)	(n = 24)
Flavored cigarettes, n (%)	12 (48.0)	13 (54.2)
Clove cigarettes, n (%)	7 (28.0)	6 (25.0)
Narghile, n (%)	5 (20.0)	7 (29.2)
Electronic cigarettes, n (%)	2 (8.0)	0 (0.0)

The proportion of women reporting wheezing was higher among the smokers than among the former smokers and never-smokers (87.5% vs. 12.5% and 0%, respectively; p = 0.030). In addition, a history of hypertension was observed only among the smokers and former smokers, of whom 14 (55.5%) and 11 (44.5%), respectively, reported having received such a diagnosis. Among women whose HADS scores were suggestive of anxiety or depression (Table 3), the majority were smokers. Five (18.4%) of the 25 smokers reported that they continued to smoke because of anxiety, whereas 3 (12.2%) reported smoking for pleasure.

**Table 3 -:** Levels of anxiety and depression among pregnant women, according to the Hospital Anxiety and Depression Scale scores, by smoking status.

Variable	Total	Smokers	Former smokers	Never-smokers
	(n = 61)	(n = 25)	(n = 24)	(n = 12)
Anxiety, n (%)				
Possible^a^	11 (18.0)	5 (20.0)	5 (20.8)	1 (8.3)
Probable^b^	13 (21.3)	7 (28.0)	2 (8.3)	4 (33.3)
Depression, n (%)				
Possible^a^	8 (13.1)	3 (12.0)	2 (8.3)	2 (16.7)
Probable^b^	5 (8.2)	4 (16.0)	0 (0.0)	1 (8.3)

a Hospital Anxiety and Depression Scale score of 8-10.

b Hospital Anxiety and Depression Scale score of 11-21.

Our findings related to how much knowledge pregnant women possess about the adverse health consequences of smoking were disappointing (Table 4). Most of the pregnant smokers and former smokers were unaware of the hazards that smoking poses to fetuses and newborn infants, as well as of tobacco-related diseases in general.

**Table 4 -:** Knowledge of and beliefs about the health consequences of smoking for maternal, fetal, and neonatal outcomes, on the part of pregnant smokers and former smokers.

Variable	Smokers	Former smokers
	(n = 25)	(n = 24)
Maternal health		
Lung disease, n (%)	6 (24.0)	7 (29.2)
Abortion/placental damage, n (%)	2 (8.0)	6 (25.0)
Cardiovascular disease, n (%)	1 (4.0)	1 (4.2)
Cancer, n (%)	1 (4.0)	0 (0.0)
No problems, n (%)	15 (60.0)	10 (40.6)
Fetal/neonatal health		
Lung disease (%)	9 (36.0)	5 (20.8)
Gestational problems,* n (%)	6 (24.0)	6 (25.0)
No harm to the fetus/neonate	10 (40.0)	13 (54.2)

* Prematurity, malformation, or low birth weight.

**Figure 1 -:**
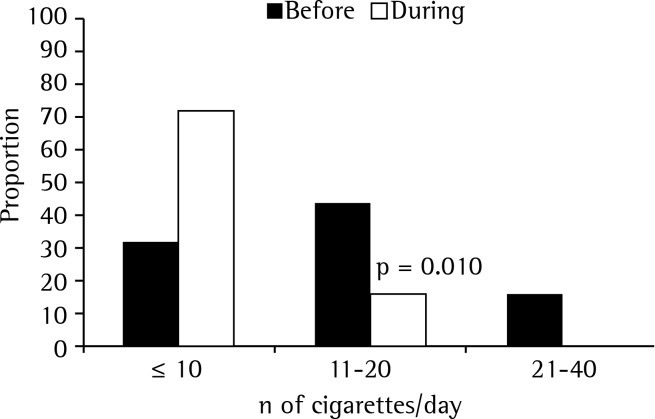
Daily consumption of cigarettes before and during pregnancy among expectant mothers in the city of Botucatu, Brazil (n = 49).

Regarding the motivational stage of change, we found that the proportion of smokers in the pre-contemplative stage was higher than was that of those in the contemplative stage (68% vs. 32%; p = 0.024). According to the results of the Fagerström Test for Nicotine Dependence, 15 (60%) of the smokers and 14 (58.3%) of the former smokers had a low degree of nicotine dependence. However, 12 (49%) of the smokers cited nicotine dependence as the main motivator for their continued smoking. We identified a reduction in smoking during pregnancy, in comparison with smoking before pregnancy (Figure 1). Of the 49 women with a history of smoking, 38 (77.5%) reported an increased desire to quit during pregnancy. Of the 24 former smokers, 12 (50%) reported that they had stopped smoking because they were pregnant. It is of note that 38 (77.5%) of the women with a history of smoking did not know that smoking cessation treatments were available, and that 40 (65%) of the women interviewed expressed a desire to have more knowledge about the adverse health consequences of smoking. Alcohol consumption during pregnancy was reported by a considerable proportion of the women with a history of smoking (42.9%), and most (85.7%) of those women reported an increase in cigarette consumption when drinking.

In our sample, the electronic media available to pregnant women included video players (in 36.1%), radio (in 62.3%), and television (in 85.2%). Television was also the medium most preferred for relaxation/enjoyment (by 49.2%). Internet use was still somewhat limited in this population, being cited by only 21 (34.4%) of the 61 women interviewed.

## Discussion

The main findings of the present study were that, among the pregnant women evaluated, there was a high prevalence of active and passive smoking during pregnancy; considerable usage of alternative forms of tobacco consumption during pregnancy; high levels of alcohol consumption during pregnancy; and limited knowledge of the adverse health consequences of smoking, including pregnancy outcomes for the fetus and newborn infant. On the basis of the preferences expressed by the interviewees, television appears to be the medium most well suited to delivering educational materials to this population (pregnant women).

We found a high (40.9%) prevalence of active smoking among pregnant women, and 72% of the pregnant smokers also reported being exposed to secondhand smoke during pregnancy. The prevalence of active smoking was higher than the 19.2-23.3% reported in the national and international literature.^(^ 1 ^)^ It should be borne in mind that this study was not designed to determine the prevalence of smoking among pregnant women in general. In fact, some of the participants were recruited from a university hospital with a prenatal outpatient clinic primarily targeting women with high-risk pregnancies, which can be associated with other disorders that predispose to increased smoking. To our knowledge, there have been no previous studies evaluating the prevalence of smoking in this subgroup of pregnant women. However, this could explain, at least in part, the high prevalence of current smoking in our study sample and indicates the need to develop prevalence studies and offer individualized smoking cessation programs at such clinics. The prevalence of passive smoking in our sample (63.9%) is comparable to the 39.2-56.7% reported in other studies of pregnant women.^(^ 8 ^,^ 12 ^)^

Our results show that a high proportion of pregnant former smokers lived with a never-smoker husband, which is consistent with the findings of previous studies showing a tendency for pregnant women to stop smoking if they have a husband who has never smoked.^(^ 13 ^)^ In addition, according to data in the literature and from the present study, pregnancy promotes smoking cessation, and that window of opportunity is not being taken advantage of, as evidenced by the fact that 77.5% of the smokers and former smokers interviewed in our study did not know that smoking cessation treatment was available.^(^ 14 ^)^ Furthermore, at least two studies have reported that mothers feel guilty about smoking during pregnancy.^(^ 14 ^,^ 15 ^)^ Therefore, a carefully presented appeal to consider smoking cessation treatment before or during pregnancy could be an effective strategy targeting this population of women. 

We found that a high proportion of the pregnant women interviewed smoked flavored cigarettes or consumed tobacco in other alternative forms. The prevalence of those forms of tobacco consumption among pregnant women in Brazil remains unknown. Like traditional (cigarette) smoking, alternative forms of tobacco consumption have adverse health consequences.^(^ 16 ^)^ Therefore, smoking cessation interventions should include information about alternative forms of tobacco use, and that information needs to be widely disseminated.

Of the pregnant women interviewed in the present study, fewer than 30% were aware of the effects that smoking has on respiratory health, and a similar proportion knew that smoking during pregnancy could lead to spontaneous abortion or damage the placenta. The consequences of maternal smoking for the fetus and newborn infant, such as prematurity, malformation, and low birth weight, were also known by only approximately 30% of the participants. Unfortunately, 40% of the smokers and 54.2% of the former smokers believed that smoking did not cause harm to the fetus or newborn infant. In addition, fewer than 6% of the women we interviewed were aware of the fact that smoking is associated with cardiovascular disease and cancer. These findings are consistent with those of studies showing that pregnant women possess only superficial knowledge of the health consequences of smoking during pregnancy.^(^ 12 ^,^ 15 ^)^

We found that the pregnant women evaluated in the present study presented with certain risk factors for smoking.^(^ 12 ^,^ 13 ^)^ The majority had a low level of formal education, and alcohol consumption during pregnancy was common (reported by 42.9%). Freire et al.^(^ 17 ^)^ also identified alcohol consumption during pregnancy in 31.3% of the pregnant smokers they evaluated. Similarly, Kroef et al.^(^ 18 ^)^ showed that, among pregnant women, smokers and former smokers consumed more alcohol than did never-smokers. In the present study, 39.3% of the pregnant women evaluated had scores of the HADS that were suggestive of anxiety and 21.3% had HADS scores suggestive of depression. Anxiety disorders are common during pregnancy,^(^ 19 ^,^ 20 ^)^ and depression has been associated with difficulty in quitting smoking among women, a situation than can be compounded by pregnancy. ^(^ 19 ^,^ 21 ^)^ Park et al.^(^ 21 ^)^ and Solomon et al.^(^ 22 ^)^ both demonstrated that, in women who spontaneously stopped smoking during pregnancy, there was a positive association between depressive symptoms at the end of pregnancy and relapse to smoking in the postpartum period.^(^ 21 ^,^ 22 ^)^

Among the pregnant women evaluated in the present study, television, the internet, and radio were the most widely used forms of electronic media, television being the form preferred by the largest proportion of the participants. Studies have shown that sending text messages (via mobile phone) advising pregnant women to quit smoking is an effective way to encourage smoking cessation during pregnancy, as is promoting interaction among pregnant smokers via online social networks.^(^ 23 ^,^ 24 ^)^ However, Bot et al.^(^ 25 ^)^ evaluated the differences in internet usage by pregnant women with different levels of education and found that those who had a low level of education were less interested in receiving e-mails about health issues than were those who had a higher level of education. In view of our finding that 65% of the pregnant smokers in the present study expressed a desire to increase their knowledge of the health consequences of smoking, we believe that educational interventions might be beneficial to these smokers.

In conclusion, the results of our study indicate that there is a high prevalence of active and passive smoking among pregnant women and that such women have limited knowledge about the health consequences of smoking, during pregnancy and otherwise. Pregnancy provides a window of opportunity for promoting smoking cessation and should be viewed as an excellent opportunity to provide more information about the health hazards of smoking and to offer smoking cessation treatment to pregnant women. Educational programs that include information about the consequences of all forms of tobacco use, employing new and effective formats tailored to this particular population, should be developed. Despite large-scale media campaigns, the level of knowledge among pregnant women regarding the long-term consequences of tobacco use remains low, and the use of alternative forms of tobacco is high among such women. However, pregnant women expressed interest in learning more about the subject, and television might be a useful tool for delivering information to this population in an attractive and continuous manner.

Potential limitations of the present study include the small sample size and the fact that it was conducted in only one city. In addition, the study sample comprised high-risk pregnant women. Therefore, the results cannot be extrapolated to pregnant women in general or to other regions of Brazil. However, because all of the women invited to participate in the study agreed to be included, there was no selection bias.

We believe that our study has added to the body of information supporting the development and implementation of new tools to improve the treatment of smoking during pregnancy. The use of material tailored to pregnant smokers and delivered in the form of videos shown or distributed to pregnant women during prenatal visits to outpatient clinics might promote smoking prevention and cessation among such women.
